# Pattern, frequency and causes of dental extraction among children/adolescents Syrian refugees: an observational study

**DOI:** 10.1186/s12887-022-03162-z

**Published:** 2022-02-21

**Authors:** Nesreen A Salim, Faleh A Sawair, Fatima Hafedh Meyad, Julian D Satterthwaite, Ashraf Abukaraky, Samiha Sartawi

**Affiliations:** 1 Prosthodontic department, School of Dentistry, Consultant in fixed and removable prosthodontics, The University of Jordan, The University of Jordan Hospital, Amman, Jordan; 2 Department of Oral and Maxillofacial Surgery, Oral Medicine and Periodontology, School of Dentistry, The University of Jordan, Jordan University Hospital, Amman, Jordan; 3 Healthcare management master student, Bahcesehir University, Bahcesehir, Turkey; 4 Professor of Restorative Dentistry, Division of Dentistry, School of Medical Sciences, University of Manchester, Oxford Road, M13 9PL Manchester, United Kingdom; 5 Department of Oral and Maxillofacial surgery, School of Dentistry, The University of Jordan, Jordan University Hospital, Amman, Jordan; 6 Prosthodontic department, School of Dentistry, consultant in fixed and removable prosthodontics, The University of Jordan, Jordan University Hospital, Amman, Jordan

**Keywords:** Dental caries, Reason, Trauma, Tooth extraction, Syrian refugees, Oral health, Zaatari camp

## Abstract

**Background:**

The Syrian conflict has had a massive impact on the dental health of refugees. Dental extraction is a good indicator of socioeconomic position and degree of oral hygiene, however there is a scarcity of evidence in the scientific literature that characterizes the reasons for extraction in refugees.

**Aims and methods:**

The current study looked at the extraction causes and related sociodemographic variables of 322 Syrian refugees (46.3% females, 53.7% males) who were treated in a dental clinic in Zaatari camp (Jordan), from September to December 2019. All child Syrian refugees (aged 4–16) visiting the facility were eligible to participate. A validated semi-structured survey was used to collect clinical and sociodemographic data from the research sample. Chi-square test, Independent sample t-test, and ANOVA test were used to examine associations between the different variables. The significance level was set at *P* < 0.05.

**Results:**

The total number of teeth extracted was 397: 25 (6.3%) permanent teeth, 371 (93.5%) primary teeth, and one mesiodens (0.2%). Overall, lower teeth were most commonly extracted (56.9%). The most common teeth that required extraction were the lower primary molars, with lower left primary second molars being the most commonly extracted (15.9%). As the level of parental education increased, the mean number of extracted teeth decreased (*P* = 0.035), additionally, as the frequency of toothbrushing increased extractions due to caries decreased significantly (*P* = 0.027).

**Conclusions:**

Dental caries and pulpal diseases were discovered to be the most prevalent causes for primary and permanent tooth extraction, with no difference between males and females. The lower left primary molar was the most commonly afflicted tooth.

## Background

Syria’s civil conflict has resulted in one of the world’s worst humanitarian and refugee crises. Since the start of the Syrian crisis in 2011, about 5.6 million Syrians have been displaced and moved to Lebanon, Turkey, Jordan, and other countries. Jordan, a poor nation with limited resources, has the world’s second greatest number of Syrian refugees (670,637 refugees) [1]. Nearly 83% of Jordan’s refugees reside in cities, while the other 17% dwell in three camps: Zaatari, Azraq, and the Emirati-Jordanian camp [2]. Zaatari camp is Jordan’s largest refugee camp, housing 79,793 people. Approximately half of the inhabitants in Zaatari camp are children, with the bulk of them being under the age of 11 (nearly 31,560) and one-fifth being under the age of 5 [1]. Currently, 19,243 children (49.% girls, 51% boys) are enrolled in formal schools from an eligible population of 25,402 (6–18 yrs), with a total of 27 schools in the camp [3].

Children of refugees are more likely to have poor physical, emotional, and social health, and their parents have restricted access to essential healthcare services [4–6]. Dental caries is one of the most prevalent childhood illnesses, affecting 60–90% of children worldwide [7]. Infection and pain associated with caries can have a significant influence on a child’s general well-being, affecting sleeping, development and behavior [8, 9], and poor oral health has a detrimental influence on the quality of life of Syrian refugees in Azraq camp in Jordan [10]. Refugees avoid regular dental visits due to low finances and a lack of insurance services [4, 5], and as a result, the pattern of dental care becomes focused on basic dental procedures with few or no preventative measures [4, 11].

The majority of early research on refugee oral health was in wealthy nations with well-established healthcare systems, and highlighted a significant burden of oral illnesses in refugees compared to the host populations [12, 13]. There has been little research on the oral health of refugees in poor countries, although recent studies have found a high frequency of dental caries and unmet dental care in a sample of Syrian youngsters at Zaatari camp in Jordan [4, 6].

The available information regarding the pattern of dental care offered to Syrians in refugee camps shows that dental extraction is the most needed/provided dental treatment (55%) and the majority of extractions was due to dental caries [4]. Restorative treatment (fillings) was the next most common provided treatment (25%) with endodontic treatment being the least, with very limited provision of procedures such as stainless steel crowns and a complete lack of more specialized treatment such as space maintainers and orthodontic treatment [4, 14]. There is also a lack of knowledge and awareness, where refugees do not seek dental treatment unless they suffer pain with very weak attendance (0.77%) for routine dental checkup [4]. The low economic status for this population (who cannot afford private dental care) necessitates using public sector dental clinics for dental care, with unavailability of advanced dental services, long waiting times, and difficulty of access to dental services all being considerable barriers to care [4, 5].

Dental services are significantly restricted in the camp, which has only two main centrally funded dental centers (with two dental clinics in each), and two further centers which are totally dependent on the voluntary contribution of national and international doctors. Continuous funding from NGOs is needed to provide treatment at no charge to the refugees. Advanced care and typical private or insurance-based services are not available.

An assessment of tooth loss in a specific population is essential for evaluating the adequacy and the availability of dental care and attitudes towards tooth extraction, as well as being important for the establishment of oral health programs. In order to develop future strategies for a reduction in tooth loss, it is important to understand the factors which lead to tooth loss, including analysis by tooth type and the relative contributions of caries, periodontal disease, trauma, and orthodontic considerations [15].

The aim of the study was to develop understanding of the dental requirements of child Syrian refugees to assist the development of targeted oral health promotion initiatives. The objectives of this study were to investigate the extraction causes in child Syrian refugees in Jordan, and explore relationships to socioeconomic variables (parental education level, medical status, gender) and oral hygiene.

## Materials and methods

### Ethical approval 

The research protocol was approved by the Ethics Review Committee of the Faculty of Dentistry of the University of Jordan (75/2020/71) and in full accordance with the world medical Declaration of Helsinki. Written informed consent was obtained from all participants’ parents.

### Study group and design

From September to December 2019, a cross-sectional clinical survey was performed. Participants comprised 322 Syrian refugees aged 4-16 who were registered as refugees in Jordan and living in the Zaatari camp. During this time, all children/adolescents who went to the Zaatari camp dental clinic for extractions were encouraged to take part. Zaatari dental clinics consists of 4 dental clinics and facilities. The treating team comprised 2 volunteering specialists, each designated to work in 1 of the 4 available clinics. Each was assisted by an intern training dentist. All services were free of charge, funded by a NGO.

### Data collection 

A structured questionnaire was used for data collection. Parents who agreed for their children to participate were interviewed, and participating children/adolescents were examined. For each patient, demographics (age, gender, parental education, duration of stay in the camp), medical history and frequency of tooth brushing were recorded; clinical variables (teeth to be extracted, reason for tooth extraction) were also recorded.

### Statistical analysis

Statistical analysis was performed using SPSS for Windows release 16.0 (SPSS Inc., Chicago, IL, USA). Descriptive statistics were generated and Chi-square test was used to examine the associations between the demographic variables and the extracted tooth type, demographic variables and reasons for extractions, and reasons for extractions with tooth type. Independent sample t-test, and ANOVA test were used to examine associations between the demographic variables and the number of teeth extracted or between age and extracted tooth type or reason for extraction. The significance level was set at *P* < 0.05.

## Results

A total of 322 children (46.3% females, 53.7% males) attended for tooth extraction. The sociodemographic characteristics of the participants are shown in Table 1. The mean age was 9.51 ± 2.32 years, with a mean duration of stay in the refugee camp of 6.83 ± 1.11 years. Only 21.1% practiced daily toothbrushing. The majority of parents had primary school education.

**Table 1:** Sociodemographic characteristics of the studied sample

Variable		Number (%)
Gender	Male	173 (53.7)
	Female	149 (46.3)
Age (year)	Mean	9.51
	Median	10
	Standard deviation	2.32
	Range	4–16
Education	None	22 (6.8)
	Primary school	292 (90.7)
	High school	8 (2.5)
Duration of stay in camp (years)	Mean	6.83
	Median	7
	Standard deviation	1.11
	Range	1–9
Medical status	Fit	310 (96.3)
	Chronic disease	12 (3.7)
Toothbrushing	Never	173 (53.7)
	Rarely	29 (9.0)
	Once a month	12 (3.7)
	Once a week	40 (12.4)
	Once a day	50 (15.5)
	Twice a day	18 (5.6)

The total number of teeth that needed to be extracted was 397: 371 (93.5%) primary teeth, 25 (6.3%) permanent teeth, and one mesiodens (0.2%). Overall, lower teeth were the most commonly extracted teeth (56.9%): 50.9% were on the right side. Most children (78.3%) had one tooth extracted, 20.2% two teeth, and 1.5% three teeth (mean 1.23 ± 0.46). In relation to the level of parental education, the mean number of teeth extracted was 1.38 ± 0.52 in children whose parents have no school education, 1.25 ± 0.47 in those with primary school parental education, and 1.00 ± 0.00 in those with high school parental education (*P *=0.035). In addition, the number of teeth extracted was significantly higher in children with chronic diseases (1.50 ± 0.52) compared with medically fit children (1.22 ± 0.45) (*P *= 0.04). No statistically significant associations were found between the number of teeth extracted and age, gender, duration of stay in camp, or frequency of toothbrushing.

The most common teeth that required extraction were the lower primary molars, with lower left primary second molars being the most common (15.9%). The least common primary teeth that required extraction were the incisors (lower right primary lateral incisors: 0.3%).

The mean age for extraction of primary incisor teeth was 7.79 ± 1.27 years, and for primary canines 11.27 ± 2.47 years, primary molars 9.34 ± 2.19, and permanent teeth 11.61 ± 2.52 (*P *< 0.001). No statistically significant associations were found between the type of extracted teeth and sociodemographic variables (other than age). The most common teeth types extracted at ages 4-6, 7-11, and 12-16 years are shown in Table 2 (*P *< 0.001). The reasons for extractions were dental caries (51.4%), dental abscess (23.4%), orthodontic reasons (7.1%), systemic diseases (6.8%), primary tooth retention (5.3%), periodontal problems (3.0%), physiological mobility (2.8%), and other reasons (0.3%). As the frequency of toothbrushing increased extractions due to caries decreased significantly (*P *=0.027). The reason for tooth extraction was not affected significantly by gender, age, medical history, or duration of stay at camp.

**Table 2:** The most common teeth types extracted at the various age groups

Age group (years)	Tooth type n (%)				
	Primary incisors	Primary canines	Primary molars	Permanent teeth	*P* value*
4–6	8 (19.5)	0	33 (80.5)	0	< 0.001^$^
7–11	20 (7.0)	10 (3.5)	243 (85.0)	13 (4.5)^a^	
12–16	0	14 (20.0)	43 (61.4)	13 (18.6)	

*P value of Chi Square test

^$^*P* = 0.019 (age 4–6 with age 7–11), *P* < 0.001 (age 4–6 with age 12–16 and age 7–11 with age 12–16). ^a^one mesiodens.

Significant differences in the reasons for extraction were noticed according to tooth type as shown in Table 3 (*P *<0.001). The extraction of primary incisors was found mostly in children with systemic disease (Diabetes mellitus) and chromosomal disease (Down’s syndrome) followed by primary tooth retention; for primary canines most common was primary tooth retention followed by dental caries; and for primary molars and permanent teeth most common was dental caries followed by dental abscesses. Significant differences in the reason for tooth extraction were noticed between teeth on the right side compared with teeth on the left as shown in Table 4 (*P *=0.028), and between the upper and lower teeth (*P *=0.004).

**Table 3:** Reasons for tooth extraction according to tooth type

Tooth type	Reasons for extraction n (%)								
	Caries	Abscess	Orthodontic reasons	Primary tooth retention	Periodontal problems	Physiological mobility	Systemic disease	Others	*P* value*
Primary incisors	1 (3.6)	1 (3.6)	1 (3.6)	5 (17.9)	3 (10.7)	4 (14.3)	13 (46.4)	0	0.001^$^
Primary canines	7 (29.2)	1 (4.2)	0	9 (37.5)	3 (12.5)	0	4 (16.7)	0	
Primary molars	177 (55.5)	85 (26.6)	27 (8.5)	6 (1.9)	6 (1.9)	8 (2.5)	10 (3.1)	0	
Permanent teeth	18 (69.2)	7 (26.9)	0	0	0	0	0	1 (3.8)^a^	

*P value of Chi Square test. ^a^Mesiodens. Orthodontic reasons: children referred by orthodontists from outside the camp.

^$^*P* = 0.019 (primary incisors with primary canines), *P* < 0.001 (primary incisors with primary molars), *P* < 0.001 (primary incisors with permanent teeth), *P* < 0.001 (primary canines with primary molars), *P* < 0.001 (primary canines with permanent teeth), *P* = 0.013 (primary molars with permanent teeth).

**Table 4:** Reasons for tooth extraction according to tooth location (side and jaw)

Location		Reasons for extraction (%)								
		Caries	Abscess	Orthodontic reasons	Primary tooth retention	Periodontal problems	Physiological mobility	Systemic disease	Others	*P* value
**Side**	Left side	99 (49.0)	47 (23.3)	14 (6.9)	16 (7.9)	7 (3.5)	2 (1.0)	17 (8.4)	0	0.028
	Right side	104 (53.3)	47 (24.1)	14 (7.2)	4 (2.1)	5 (2.6)	10 (5.1)	10 (5.1)	1 (0.5)	
**Jaw**	Upper teeth	85 (49.7)	33 (19.3)	8 (4.7)	14 (8.2)	6 (3.5)	5 (2.9)	19 (11.1)	1 (0.6)	0.004
	Lower teeth	118 (52.2)	61 (27.0)	20 (8.8)	6 (2.7)	6 (2.7)	7 (3.1)	8 (3.5)	0	

*P* value of Chi Square test

## Discussion

The current study provided insight into the patterns and reasons for extraction in a refugee child population. Significant variations in the type and position of extracted teeth were found, as well as significant variations in the reasons and type of removed teeth from both dentitions and age groups.

The prevalence of extractions and the reasons for extraction may provide insight regarding the availability of dental treatment, the prevalence of dental care, and attitudes about tooth extraction. Despite the fact that the frequency of dental caries in young children has reduced significantly in recent years [15], caries continues to afflict many children in the general population, particularly in developing nations and refugees where illness is on the rise [4, 6, 16]. Despite the relative large quantity of research detailing the causes for permanent tooth extraction [17–20], little information is available regarding the reasons for primary tooth extraction and the tooth type removed in refugee children.

The current study was the first to look into the patterns of dental extraction and relationship to socioeconomic variables on tooth loss among Syrian children in a large refugee camp in Jordan. Understanding the pattern of dental care gives insight into the dental needs and oral health of a population [4, 11, 21], and an understanding of the causes for tooth loss as well as the relative contributions of caries, periodontal disease, trauma, and orthodontic concerns will help inform future plans for meeting dental needs and planning preventive programs to reduce tooth loss. Furthermore, the current study findings will aid in the development of a specialized oral health promotion program for Syrian children living in refugee camps with limited resources.

According to the findings of this study, the primary reason for extraction is caries and its sequelae (75%): caries as a reason for extraction was reported only when the tooth was not restorable (i.e. not including teeth that were retained and close to exfoliation that were extracted due to retention). This finding is consistent with previous research, which found that dental caries was the most prevalent cause of early primary tooth loss [4, 6, 15, 22]. However, our study reported a higher percentage of extractions as a result of caries than previously reported, which may be due to different levels of nutrition, socioeconomic variables, dental awareness, and water fluoridation in these nations [23]. A common misconception among parents is that primary teeth do not require treatment since new teeth would emerge later; this reflects parental lack of information and poor attitudes toward preventive and primary dental care for primary teeth [4, 24]. For example, previous refugee research has shown that a significant percentage of extractions are performed at the request/insistence of parents, even though the tooth might be restorable [4].

Children in the current study spent an average of 7 years in the camp. This implies that a substantial number of the children were either born in the camp or came there within the first few years of their lives. Unfortunately, the camp's water supply is not fluoridated, [25] which may partly explain caries as the main reason for extraction in this group, along with nutrition, socioeconomic variables, and degree of dental awareness. It is worth mentioning that community-based water or salt fluoridation initiatives have yet to be established in Syria [26].

Dental extractions owing to retention of teeth were more prevalent than extractions due to mobility, this is in line with an Indian study [22] although the reverse has also been shown [15]. The latter trial also reported a substantially higher number of orthodontic extractions, perhaps highlighting the lack of information and awareness of the present study population group, as well as the paediatric population's underutilization of oral health services. A large number of patients from the camp plainly demonstrate a severe lack of dental care in this region, especially orthodontic treatment [14]. Periodontal disease accounted for 3 % of tooth extractions in the current study, compared to 0.45 % in a study conducted in southwest Nigeria [27], probably reflecting poor oral health and refugees' propensity to seek dental care late in the course of disease, when tissue damage would have been progressed, needing extraction.

Interestingly, the number of teeth extracted was significantly higher in children with chronic diseases compared with medically fit children. It has been reported that many systemic conditions (such as neutropenia, leukocyte disorders, Down’s syndrome, diabetes mellitus and Papillon-Lefe`vre syndrome) may reduce the host response in children and adolescents, thus increasing their susceptibility to periodontal bone loss and ultimately loss of teeth [28], and this may explain the higher rates of extraction in this group.

Regarding the distribution of dental extraction by tooth type, primary lower molars were the most commonly extracted teeth (primarily due to caries). This finding is in line with many previous studies [4, 15, 22], and has been reported to be due to easier food collection in the lower jaw and the contextual unfavourable position of primary molars, which makes plaque removal more difficult. Furthermore, the pits and fissures on the chewing surfaces of primary molars create an ideal habitat for Streptococcus mutans to adhere [29]. Another cause might be anatomical diversity of posterior teeth, accessibility, brushing dexterity, higher frequency of sweet-eating/snacking, and hereditary vulnerability to caries [4]. This study discovered a greater percentage of extraction of primary second molars (33.1%) than primary first molars (28.1%), which is inconsistent with previous reports [29, 30]. This is a critical finding as early loss of primary second molars is a concern since these teeth act as a guide for emerging permanent first molars, and their removal might create problems, as their absence may result in a decrease in arch length due to mesial migration of permanent molars [31, 32]. Also of note is that in our study the main reason for removal of other primary teeth was retention rather than caries.

There were no statistically significant variations in the primary tooth type removed based on gender. Previous studies observed a similar observation [15, 22]. The positive impact of parental education on the pattern of extraction has also been reported in previous work, highlighting that greater education levels are related with a better knowledge of oral and general health and a reduced rate of dental caries [21, 33].

As the frequency of toothbrushing increased, extractions due to caries decreased significantly in this study. Regrettably, the majority of children in this study showed poor oral hygiene practices, as only 21% of them brush once to twice daily. This may point to a lack of educational and preventive services as a result of inadequate finances and restricted access to timely dental care. There are several reasons why refugees may lose interest in dental care: conflicting resettlement objectives, administrative problems, language barriers, a lack of transportation, traditional health beliefs or misconceptions, and past bad health service experiences are examples of these [34, 35], although it should be noted that achieving health-related behavioural change in a complex environment (such as a refugee camp) is challenging. Dental health education and promotion programs are required to raise the knowledge and awareness of parents and their children. These programs should emphasize the importance of children's primary as well as permanent teeth, and optimal caries prevention techniques in order to avoid early loss of molars.

## Conclusion

Dental caries and pulpal diseases were discovered to be the most prevalent causes for primary and permanent tooth extraction, with no difference between males and females. The lower left primary molar was the most commonly afflicted tooth. 

## Data Availability

All collected data from patients analysed during this study are included in this published article. Some datasets are available from the corresponding author on reasonable request.

## Abbreviations

FDI: The Fédération Dentaire Internationale
MD: Mesiodens
